# Clinical significance of evaluating hormone receptor and HER2 protein using cell block against metastatic breast cancer: a multi-institutional study

**DOI:** 10.18632/oncotarget.27163

**Published:** 2019-10-01

**Authors:** Akira Matsui, Yuya Murata, Norikazu Masuda, Kiyoshi Mori, Masato Takahashi, Katsushige Yamashiro, Kenjirou Aogi, Shigeto Maeda, Masahiro Itou, Shinji Ozaki, Kazuya Kuraoka, Yasuyuki Satou, Shu Ichihara, Eriko Tokunaga, Kenichi Taguchi, Takanori Watanabe, Hiroyoshi Suzuki, Aiko Nagayama, Rieko Nishimura

**Affiliations:** ^1^ Department of Breast Surgery, National Hospital Organization Tokyo Medical Center, Meguro-ku, Tokyo 152-8902, Japan; ^2^ Department of Clinical Laboratory, National Hospital Organization Tokyo Medical Center, Meguro-ku, Tokyo 152-8902, Japan; ^3^ Department of Breast Surgery, National Hospital Organization Osaka National Hospital, Chuou-ku, Osaka-shi, Osaka 540-0006, Japan; ^4^ Department of Clinical Laboratory, National Hospital Organization Osaka National Hospital, Chuou-ku, Osaka-shi, Osaka 540-0006, Japan; ^5^ Department of Breast Surgery, National Hospital Organization Hokkaido Cancer Center, Shiraisi-ku, Sapporo-shi, Hokkaido 003-0804, Japan; ^6^ Department of Pathology, National Hospital Organization Hokkaido Cancer Center, Shiraisi-ku, Sapporo-shi, Hokkaido 003-0804, Japan; ^7^ Department of Breast Surgery, National Hospital Organization Shikoku Cancer Center, Minamiumemoto, Matsuyama-shi, Ehime 791-0280, Japan; ^8^ Department of Surgery, National Hospital Organization Nagasaki Medical Center, Oomura-shi, Nagasaki 856-8562, Japan; ^9^ Department of Clinical Research Center, National Hospital Organization Nagasaki Medical Center, Oomura-shi, Nagasaki 856-8562, Japan; ^10^ Department of Breast Surgery, National Hospital Organization Kure Medical Center, Kure-shi, Hiroshima 737-0023, Japan; ^11^ Department of Pathology, National Hospital Organization Kure Medical Center, Kure-shi, Hiroshima 737-0023, Japan; ^12^ Department of Breast Surgery, National Hospital Organization Nagoya Medical Center, Naka-ku, Nagoya-shi, Aichi 460-0001, Japan; ^13^ Department of Pathology, National Hospital Organization Nagoya Medical Center, Naka-ku, Nagoya-shi, Aichi 460-0001, Japan; ^14^ Department of Breast Surgery, National Hospital Organization Kyushu Cancer Center, Minami-ku, Fukuoka-shi, Fukuoka 811-1395, Japan; ^15^ Department of Pathology, National Hospital Organization Kyushu Cancer Center, Minami-ku, Fukuoka-shi, Fukuoka 811-1395, Japan; ^16^ Department of Breast Surgery, National Hospital Organization Sendai Medical Center, Miyagino-ku, Sendai-shi, Miyagi 983-8520, Japan; ^17^ Department of Pathology, National Hospital Organization Sendai Medical Center, Miyagino-ku, Sendai-shi, Miyagi 983-8520, Japan; ^18^ Department of Clinical Laboratory, National Hospital Organization Shikoku Cancer Center, Minamiumemoto, Matsuyama, Ehime 791-0280, Japan

**Keywords:** metastatic breast cancer, receptor discordance, cell block, cytological specimen, prognosis

## Abstract

Hormone receptor and human epidermal growth factor receptor 2 (HER2) protein tests in metastatic breast cancer tissue are recommended in the guidelines of the American Society of Clinical Oncology/American Pathology Association. As part of a multi-institutional study by the National Hospital Organization, we conducted an investigation to examine these molecular markers, using cytological specimens as a substitute for tissue specimens from breast cancer metastasis. To confirm the usefulness of receptors tested in metastatic lesions, the treatment course of registered metastatic breast cancer patients was analyzed. During the April 2015 to March 2016 registration period, there were 62 registrations. Types of metastatic lesions include pleural fluid (44 samples), ascites (14 samples), lymph nodes (2 samples), pericardial fluid (1 sample), and dorsal subcutaneous mass (1 sample). A stable test result was obtained by adopting the receptor examination method, using cell block for immunostaining cytological specimens. The discordance rates of estrogen receptor (ER), progesterone receptor (PR), and HER2 protein expression were 18.2% (95% confidence interval (CI): 7.9–28.8%), 36.4% (95% CI: 23.7–49.1%), and 8.2% (95% CI: 0.1–16.3%), respectively, between the primary tumor and metastatic lesion. Patients who changed from primary negative to metastatic positive ER status had taken a significantly longer time for metastatic foci to appear. Patients with positive ER status in metastatic lesions had significantly better prognosis than ER-negative cases (P = 0.030) by the Log-Rank test. The ER status of the metastatic lesion and the metastatic site were independent prognostic factors by Cox multivariate analysis. Receptor examination with cytological specimens in metastatic lesions has been useful as it provides guidance for the treatment of metastatic breast cancer.

## INTRODUCTION

When treating recurrent breast cancer, therapeutic agents are commonly selected on the basis of hormone receptor (HR) and human epidermal growth factor receptor 2 (HER2) protein expression. These molecular markers are well established as useful predictors of therapeutic effect in recurrent breast cancer treatment, similar to in primary breast cancer. The American Society of Clinical Oncology/College of American Pathologists (ASCO/CAP) guidelines recommended that hormone receptors and HER2 are tested in tissue specimens obtained from recurrence or metastases in breast cancer patients [1–4]. This is due to the possibility that the receptor state may change during the progress from primary to recurrent tumor [5–8].

However, the collection of tissue specimens from metastatic or recurrent foci is often difficult. Cytological analysis can be applied to metastatic lesion specimens, such as body cavity fluids and sites where tissue biopsy is difficult. Therefore, cytological diagnosis is useful for pathological diagnosis of cancer metastasis. In addition, applying cytological analysis to receptor examination may provide a useful treatment guide for a selected relapsed patient.

In order to examine the breast cancer receptor using a cytology specimen, it is necessary to prepare multiple slides containing cancer cells. For this, we adopted the cell block (CB) method. There are already some reports of receptor examination using breast cancer cytology specimens, and for hormone receptors, the agreement rate between the cytological and tissue specimen is good [9–13]. For the HER2 test, it is reported that the agreement rate with the tissue specimen is improved by adding the dual *in situ* hybridization (DISH) assay for the case of HER2 2+ by immunostaining [14–16].

In addition, receptor testing using cytological specimens can be a quick, inexpensive, and less invasive alternative when compared to methods using tissue specimens.

As part of a multi-institutional study by the National Hospital Organization, we conducted a study to examine hormone receptors using cytological specimens from breast cancer metastases.

We adopted the receptor examination method in cytological specimens using CBs unified in multiple institutions, and stable test results were obtained [17]. Furthermore, in order to confirm whether receptor assessment in breast cancer metastatic lesions is useful for determining a breast cancer treatment strategy, we analyzed the treatment course of metastatic breast cancer patients registered for this multi-institutional study.

## RESULTS

### Registered specimens

During the registration period, the number of registrations in which patient consent was obtained was 62. For these breast cancer metastasis foci, receptor examination using CB was performed. The background of the cases is shown in Table 1.

**Table 1:** Patients characteristics

Variable	Number
Age (mean ±SD)	61.7 ± 11.0
Clinical Stage at diagnosis	
I	5
II	29
III	13
IV	10
unknown	5
Metastatic lesion	
Pleural fluid	44
Ascites	14
Lymph node	2
Pericardial fluid	1
Subcutaneous metastatic nodule on the back	1
Primary lesion of breast	
Solitary	57
Synchronous multiple lesion	4
Metachronous multiple lesion	1
Time of distant metastasis	
Metachronous metastasis	56
Synchronous metastasis	6

All patients were females aged 40 to 80 years (average 61.7 years old, median 63 years old). The types of metastatic lesions were pleural effusion (44 specimen), ascites (14 specimen), lymph node (2 specimen), pericardial effusion (1 specimen), and dorsal subcutaneous mass (1 specimen).

### Difference in receptor expression between primary tumor and metastatic lesion

The status of receptor expression in the primary tumor and metastatic lesion was compiled in a paired sample (Table 2).

**Table 2:** Discordance rate of ER, PR and HER2 expression between primary and metastatic lesion

		Metastatic lesion		Discordance	(95% CI)
ER		positive	negative		
**Primary lesion**	positive	35	7		
	negative	3	10	10/55(18.2%)	(7.9–28.5%)
**PR**		positive	negative		
**Primary lesion**	positive	16	17		
	negative	3	19	20/55(36.4%)	(23.7–49.1%)
**HER2**		positive	negative		
**Primary lesion**	positive	3	3		
	negative	1	42	4/49(8.2%)	(0.1–16.3%)

In ER and PR, there was a difference in receptor expression between the primary tumor and metastatic lesion in 10 (18.2% (95% confidence interval (CI): 7.9–28.8%)) and 20 specimens (36.4% (95% CI: 23.7–49.1%)) respectively, of 55 specimens. HER2 protein expression did not agree in 4 out of 49 specimens (8.2% (95% CI: 0.1–16.3%)) between the primary tumor and metastatic lesion.

In a comparison based on four groups of primary/metastatic receptor status (positive/positive, positive/negative, negative/negative, negative/positive), patients who changed from primary negative to metastatic positive ER status had taken a significantly longer time for metastatic foci to appear (Table 3). In addition, many patients with metastatic negative ER and PR from primary positive status received hormonal therapy until registration, either as postoperative adjuvant therapy or treatment after recurrence (Table 4). Similarly, in patients with metastatic negative HER2 from primary positive status, anti-HER2 therapy was administered as a recurrence treatment before registration.

**Table 3:** Relationship between expression status of ER, PR, HER and the interval until re-examination for metastatic lesion

	Status (primary/metastatic)	Number	Interval (month)
ER	Negative/negative	10	58.7
	Negative/positive	3	187.3*
	Positive/positive	35	85.9
	Positive/negative	7	89.1
PR	Negative/negative	19	67.7
	Negative/positive	3	161.7
	Positive/positive	16	78.3
	Positive/negative	17	103.3
HER2	Negative/negative	42	87.9
	Negative/positive	1	90.0
	Positive/positive	3	35.3
	Positive/negative	3	47.3

^*^ *P* < 0.05.

**Table 4:** Relationship between expression status of ER, PR, HER and the previous treatment

	Status (primary/metastatic)	Adjuvant	For metastasis
		Endocrine treatment	Endocrine treatment
ER	Negative/negative	0/10(0%)	0/10(0%)
	Negative/positive	1/3(33.3%)	2/3(66.7%)
	Positive/positive	24/35(68.6%)	25/35(71.4%)
	Positive/negative	7/7(100%)	5/7(71.4%)
PR	Negative/negative	6/19(31.6%)	6/19(31.6%)
	Negative/positive	1/3(33.3%)	3/3(100%)
	Positive/positive	11/16(68.7%)	9/16(56.3%)
	Positive/negative	14/17(82.4%)	13/17(76.4%)
		**Trastuzumab**	**Trastuzumab**
HER2	Negative/negative	2/42(4.8%)	2/42(4.8%)
	Negative/positive	0/1(0%)	0/1(0%)
	Positive/positive	1/3(33.3%)	1/3(33.3%)
	Positive/negative	0/3(0%)	2/3(66.7%)

### Outcomes of treatment based on receptor status of metastatic lesion

**Figure 1:**
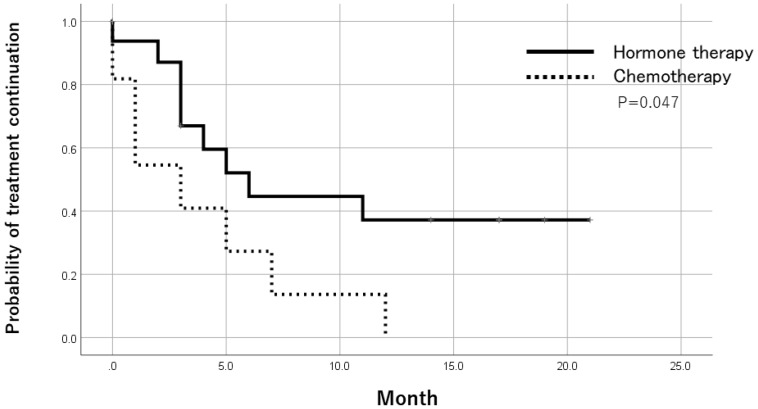
Time to treatment failure after trial registration by treatments in ER-positive patients. In ER-positive cases of metastatic lesions, the treatment period of patients receiving endocrine therapy was significantly longer than patients receiving chemotherapy as a post-registration treatment.

Among ER-positive cases in metastatic lesions, the treatment duration was significantly longer in patients who received endocrine therapy as a post-registration treatment than in those who received chemotherapy by the Log-Rank test (Figure 1).

### Receptor status in metastatic lesion and prognosis after registration

**Figure 2:**
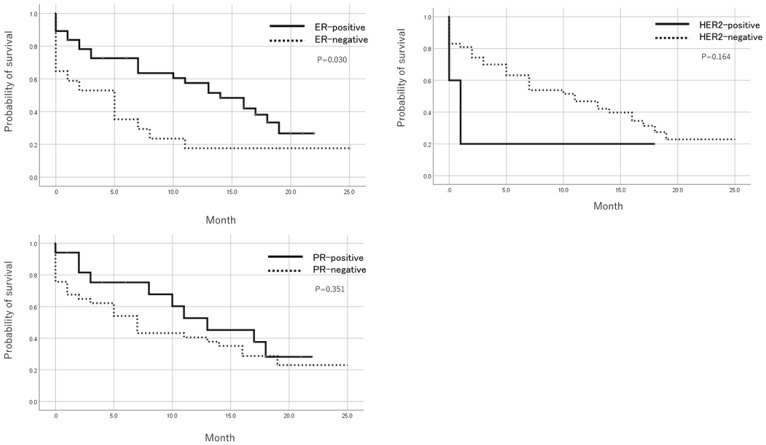
Prognosis after trial registration by ER, PR, and HER2 status. Patients with a positive ER status in metastatic foci showed a significantly better prognosis after registration than ER negative cases. There was no significant difference in prognosis based on PR and HER2 expression.

**Figure 3:**
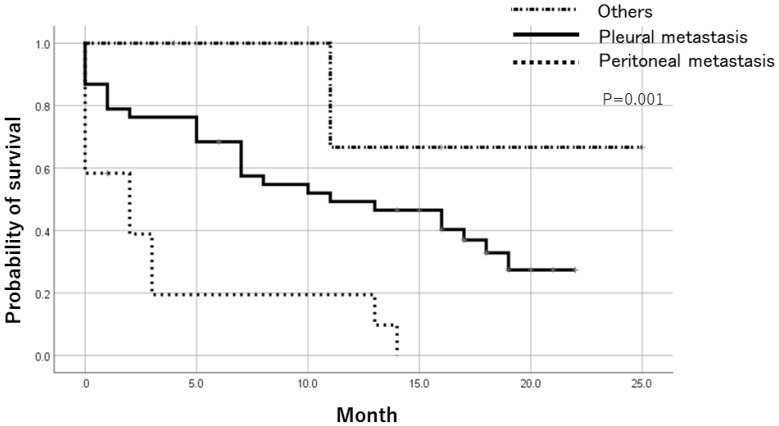
Prognosis after trial registration by metastatic lesion. Comparing the prognosis based on the registered metastatic sites, the prognosis was significantly worse in cases registered as peritoneal metastasis.

Patients with positive ER status in metastatic lesions had a significantly better prognosis than ER-negative cases after registration by the Log-Rank test. There was no significant difference in prognosis based on PR and HER expression (Figure 2). In addition, when the prognosis was compared between pleural metastasis, peritoneal metastasis, and others, the prognosis was significantly worse in cases registered as peritoneal metastasis by the Log-Rank test (Figure 3). Table 5 shows the result of Cox multivariate analysis including these factors. The ER status of the metastatic lesion and the metastatic site were independent prognostic factors.

**Table 5:** Multivariate analysis of factors affecting prognosis after registration

Factor	Hazard ratio	95% CI	*P*-value
ER	0.327	0.158–0.678	0.003
Metastatic lesion			
Pleural fluid	0.217	0.096–0.488	<0.001
Others	0.055	0.006–0.465	0.008

## DISCUSSION

The receptor examination method for metastatic breast cancer using CB was easy to introduce. In institutions conducting receptor examination of tissue specimens in daily practice, the procedure is easy and cost-effective, so there are few obstacles to its introduction. Pleural effusion (44 samples) followed by ascites (14 samples) were the most common samples in this study. By examining the receptor status on cytological specimens using CBs, treatment guides were obtained for metastatic lesions that had so far been difficult to evaluate.

In the examination of tissue specimens, it is reported that the discordance rate of receptor status in the primary tumor and metastatic lesion is 12–30% in ER, 18–42% in PR, and 5–16% in HER2 [18–21]. In our study, the discordance rate was 18.2% (95% CI: 7.9–28.8%) in ER, 36.4% (95% CI: 23.7–49.1%) in PR and 8.2% (95% CI: 0.1–16.3%) in HER2, which was comparable with the reported rate. The fact that the discordance rate was similar between the cytological and tissue specimens indicates the reliability of the receptor testing in the cytological specimens.

Although direct comparison of cytology specimens and tissue specimens from breast cancer metastatic foci will prove the reliability of cytology cell block in receptor examination, it is not easy due to the difficulty of sampling metastatic lesions. Vohra examined receptor and HER2 expression in 134 breast cancer patients, compared cytology cell block and tissue samples, and showed high agreement rates, but only 18 metastatic lesions were included [22]. Because most of our target specimens were cytological preparations from liquid samples, direct comparison with tissue samples at the metastatic site was difficult.

Focusing on changes in hormone receptor expression, it was necessary to review treatment strategies. This is because ER did not coincide with the primary tumor at 18.2% of the recurrent foci. As metastatic negative change of HRs from primary positive status may occur when receiving multiple endocrine treatments as adjuvant or recurrent therapy, it is better to reconfirm the receptor status in the metastatic lesion. The discordance rate in PR was greater, with nearly half of the cases with primary positive tumors converted negative status in metastatic lesions. PR expression is controlled by ER, and PR status is most susceptible to the influence of previous endocrine treatments [23]. Additionally, there was a positive change of ER in the metastatic site from primary negative status in a few cases, in which a long time had passed until the metastatic lesion had appeared. In general, hormone-dependent breast cancer progression is slow, and in some patients, metastasis appears over a long period of time. Assuming the heterogeneity of the primary tumor, when a small number of ER-positive clones metastasize over a long period of time, it may deemed an ER-positive conversion in metastatic lesions. In patients with a long course to metastasis, it is therefore recommended to reexamine the receptor status, considering positive change of the hormone receptor.

In metastatic ER-positive cases, the endocrine therapy treatment period after registration was significantly longer compared with that of chemotherapy. Many ER-positive patients who choose chemotherapy have more advanced disease and cannot be easily compared. In the metastatic ER-positive cases, it seems preferable to give priority to endocrine treatment when the general condition is not bad.

Furthermore, metastatic ER-positive cases have better prognosis than ER-negative cases, and it is necessary to prepare a long-term treatment plan. In a clinical setting, patients with ascites due to breast cancer metastasis often progress rapidly after metastasis. Patients with peritoneal metastasis had poor prognosis in this study, consistent with actual clinical experience. Depending on the metastatic site, it is necessary to modify the treatment policy.

Reasons for receptor examination in metastatic lesions include cases in which the primary tumor’s receptor status is unknown or there are multiple primary lesions, and thus receptor testing in metastatic lesions is inevitable. In this study, although the receptor status of the primary tumor was known, there were many patients who wanted to review the treatment strategy according to the receptor status of the metastatic lesion. In cases where the administered treatment was ineffective, a metastatic lesion receptor test was used to monitor the treatment in detail. Receptors and HER2 protein expression are often more difficult to determine in cytological specimens than tissue specimens. However, it is considered an alternative examination method in cases where biopsy cannot be easily performed. Consequently, receptor examination in cytological specimens is useful, as it provides a treatment guide in metastatic breast cancer.

## MATERIALS AND METHODS

Nine institutions belonging to the National Hospital Organization (NHO), (Hokkaido Cancer Center, Sendai Medical Center, Tokyo Medical Center, Nagoya Medical Center, Osaka National Hospital, Kure Medical Center, Nagasaki Medical Center, Kyushu Cancer Center, and the Shikoku Cancer Center), participated in the study.

The study plan was approved by the National Hospital Organization Central Ethical Review Board. The registration period was from April 1, 2015 to March 31, 2016. Patients diagnosed with breast cancer metastasis, pathological diagnosis by cytological specimens, and further referred for receptor examination were targeted. Patients signed the informed consent form and accepted the registration. In each institution, the examination of HR and HER2 protein was performed on cytology specimens obtained from breast metastases.

### Preparation of CB and staining of receptor

All participating facilities prepared CB using the same methodology. First, specimens collected from metastatic sites were fixed in 10% buffered formalin for 6 to 48 hours. Thereafter, the tube containing the sample was centrifuged at 3000 rpm for 5 minutes to remove formalin. After this, 0.5 mL of 1% sodium alginate was added, and the tube was centrifuged again at 3000 rpm for 5 minutes, after which 0.5 mL of 1 M calcium chloride was added. The gel pellets formed were embedded in paraffin to make paraffin CB. CB was then prepared in the same way for the tissue specimens.

Sections prepared from CB were stained with hematoxylin-eosin (HE) and underwent immunohistochemistry (IHC). Each facility used an autostainer for IHC staining. The HER2 DISH assay was performed using Ventana Bench Mark in the Shikoku Cancer Center. Evaluation of HR and HER2 immunostaining was performed at each institution, and evaluation of DISH was performed at the Shikoku Cancer Center.

### Evaluation of HR expression

Staining for HR was evaluated as positive or negative according to the following criteria: cases where there was any nuclear staining of tumor cells were determined to be positive, and cases where there was no nuclear staining were deemed negative. The positive rate of stained tumor cells was not considered for evaluation. For cytological specimens containing certain non-neoplastic cells, it is difficult to estimate the proportion of stained cells in tumor cells.

### Evaluation of HER2 protein expression

We scored staining results of the HER2 protein for 0, 1+, 2+, or 3+, according to the following criteria: 3+, strong staining of the cell membranes of tumor cells; 2 +, intermediate staining of the cell membranes of tumor cells; 1+, weakly incomplete cell membrane staining of tumor cells; 0, no staining. Again, the percentage of stained tumor cells was not considered for scoring. The expression status of the HER2 protein was classified as negative in samples scoring 0 or 1+, undetermined in the sample with a score of 2+, and positive in samples with a score of 3+. When the result of HER2 immunostaining was 2+, DISH was performed.

### Evaluation of results of HER2 DISH assay

The INFORM HER2/neu double *in situ* hybridization DNA probe cocktail assay was used for slides prepared from CB. The DISH assay was performed according to the manufacturer’s recommended protocol for tissue specimens. To avoid subjective bias, HER2/neu (black) and chromosome enumeration of probes 17; a CEP17 (red) ratio were manually counted by two investigators under a light microscope for each sample. At least 20 cells were counted. When the HER2/CEP17 signal number ratio was 2.0 or more, or the signal number ratio was less than 2.0 but the average number of HER2 signals per cell was 6.0 or more, it was deemed amplified.

### Discordance rate between the primary tumor and metastatic lesion

We compared the expression of HR and HER2 protein on pairs of samples whose expression status was confirmed in both the primary tumor and metastatic lesion, and calculated the concordance and discordance rate of expression. In addition, the expression of the primary/metastatic receptor status was divided into 4 groups (positive/positive, positive/negative, negative/negative, negative/positive). We compared the period and the details of previous treatment until re-examination of metastatic lesions.

### Therapeutic effect and prognosis based on receptor expression of metastatic lesion

For patients with ER-positive metastatic lesions, the duration of treatment after registration was compared between endocrine therapy and chemotherapy. We also compared the prognosis after enrollment based on receptor expression in metastatic lesions. In addition, Cox multivariate analysis was performed on prognosis after registration, including age and metastatic ER, PR, HER2 expression and metastatic site.

### Statistical analysis

SPSS Ver 25 was used for statistical analysis. For the comparison of the mean value among 4 groups, analysis of variance was used. Treatment continuation rate and survival rate were calculated by the Kaplan-Mayer method, and the difference was examined by the Log-Rank test. Multivariate prognostic analysis was performed using the Cox proportional hazards model. Significant differences were assessed at a significance level of 5%.

## Abbreviations

HR: hormone receptor
HER2: human epidermal growth factor receptor 2
DISH: dual *in situ* hybridization
ER: estrogen receptor
PR: progesterone receptor
HE: hematoxylin-eosin
IHC: immunohistochemistry
CB: cell block
CI: confidence interval
